# Autologous Peripheral Blood-Derived Stem Cells Combined with Platelet-Rich Plasma is Associated With Clinical and Radiological Improvement in Anterior Cruciate Ligament Ruptures—Preliminary Clinical and MRI Findings in 47 Consecutive Patients

**DOI:** 10.1007/s43465-026-01904-0

**Published:** 2026-07-28

**Authors:** Patrick Weninger, Andreas Herbst, Veronika Pfafl-Weninger, Celina Reitböck

**Affiliations:** Sports Medical Center, Vienna, Austria

**Keywords:** Anterior cruciate ligament, ACL rupture, Stem cell therapy, Biological ligament healing, Regenerative medicine, PRP, Ultrasound-guided injection, Sports medicine, Functional outcomes, Non-surgical ACL treatment

## Abstract

**Background:**

Anterior cruciate ligament (ACL) ruptures are common injuries in young and active individuals and can lead to long-term functional impairment and early onset osteoarthritis. While ACL reconstruction remains the standard of care, recent evidence suggests that spontaneous healing may be possible in selected cases. Regenerative and biologic treatment concepts have therefore gained increasing interest. This study investigates the clinical and radiological outcomes of a stem cell–based regenerative treatment for traumatic ACL ruptures and presents findings as preliminary observational evidence.

**Methods:**

In this single-center retrospective case series, 47 patients with isolated partial and complete traumatic anterior cruciate ligament (ACL) ruptures were treated using a biologic, non-surgical protocol. Diagnosis of an ACL rupture was confirmed by standardized clinical examination (Lachman test, anterior drawer test, and pivot shift test) and magnetic resonance imaging (MRI), which was additionally used to assess rupture morphology and synovial sheath integrity. All patients underwent an ultrasound-guided injection of autologous biologic products derived from peripheral venous blood, consisting of 8 mL of an autologous blood-derived stem cell preparation (BSC), comprising approximately 2–3 mL of concentrated mononuclear cells suspended in 6 mL of autologous platelet-containing plasma, injected directly into the torn ACL. Patients were allowed immediate full weight-bearing without bracing and followed a standardized physiotherapy program. Clinical follow-up was conducted for 12 months, including assessment of pain (VAS), functional outcomes (IKDC, Lysholm), return-to-sport timelines, and MRI-based evaluation of ligament morphology.

**Results:**

The mean age of the cohort was 28.1 years (95% CI 26.0–30.2). Functional outcomes improved continuously throughout follow-up, with mean IKDC and Lysholm scores of 93.6 (95% CI 92.4–94.8) and 94.0 (95% CI 92.7–95.3), respectively, at 12 months. Pain levels decreased markedly, reaching a mean VAS score of 0.36 (95% CI 0.19–0.53). Patients returned to running after a mean of 2.19 weeks (95% CI 1.93–2.45), and most resumed their pre-injury activity level by one year. No infections or re-ruptures occurred and only one secondary ACL reconstruction was recorded. Patients with an intact synovial sheath demonstrated faster return to pivoting sports (26.8 vs. 33.2 weeks) compared with patients with a complete ACL rupture. MRI at 3 months showed evidence of improved ligament morphology in all but one patient.

**Conclusions:**

This preliminary case series suggests that stem cell–based biologic stimulation may be associated with functional recovery and radiological improvement in selected patients with ACL ruptures. Prospective controlled trials are required to validate these findings.

## Introduction

The anterior cruciate ligament (ACL) is essential for maintaining knee joint stability and plays a pivotal role in controlling anterior tibial translation and rotational stability. Ruptures of the ACL are among the most frequent severe knee injuries, predominantly affecting young and physically active individuals engaged in pivoting or contact sports [1]. The true incidence across the European Union (EU) remains uncertain, as no EU-wide registry data are available. However, population-based data from Sweden report an annual Anterior Cruciate Ligament Reconstruction (ACLR) of up to 32 per 100,000 inhabitants [2]. When extrapolated to the EU population, this would correspond to an estimated 140.000 ACLR annually.

Given its demographic and lifestyle relevance, ACL rupture remains a major topic of orthopedic and sports medicine research. The injury often occurs in otherwise healthy and active individuals, and the long-term sequelae—including chronic instability, functional impairment, and early onset of osteoarthritis—can significantly affect patients’ lifestyle, participation in sports, and overall quality of life [2, 3]. Even after successful surgical reconstruction, persistent quadriceps weakness, proprioceptive deficits, and degenerative changes are frequently reported [3, 4].

Over the past decades, the gold standard for ACL management has been surgical reconstruction, aiming to restore mechanical stability rather than biological healing. However, recent evidence indicates that spontaneous healing of the ACL may be possible under optimized biological conditions, challenging the long-standing paradigm that complete ruptures cannot heal [5]. These findings, together with the limitations of reconstructive surgery—including donor-site morbidity, tunnel widening, and graft failure—have led to growing interest in regenerative and biologic approaches to ACL repair [6–8].

Among these approaches, mesenchymal stem cells (MSCs) have emerged as a promising therapeutic option due to their differentiation potential, paracrine effects, and ability to modulate inflammation and promote tissue regeneration [9, 10]. Several preclinical and early clinical studies have demonstrated that MSCs can enhance ligament healing by promoting collagen synthesis, vascularization, and restoration of native tissue architecture [11, 12]. Recently, first-in-human trials have reported encouraging outcomes for biologic ACL repair and MSC-augmented scaffolds, indicating equal structural integrity and functional recovery compared with standard operative treatment [13, 14].

Platelet-rich plasma (PRP) has gained increasing attention as an adjunctive biologic treatment due to its high concentration of growth factors and cytokines, including platelet-derived growth factor, transforming growth factor-β, and vascular endothelial growth factor, which are known to support tissue repair processes. Experimental and clinical studies suggest that PRP may exert anti-inflammatory, chemotactic, and proliferative effects, potentially enhancing collagen remodeling and cellular recruitment within injured ligament tissue [15–17]. Preparation parameters such as centrifugation speed, platelet yield, and leukocyte content substantially influence the cellular composition and biological activity of PRP preparations [18]. Conservative treatment with PRP alone has also been evaluated in highly active patients with acute ACL injuries, with promising early results [19].

Although PRP has not yet been established as a stand-alone treatment for complete ACL ruptures in clinical practice, accumulating experimental and translational evidence indicates that PRP provides a biologically active microenvironment rich in growth factors and cytokines that can enhance ligament healing processes. In this context, PRP appears to act synergistically with mesenchymal stem cell–based therapies by promoting cell recruitment, modulating inflammation, and supporting extracellular matrix remodeling within injured ligament tissue [17].

Importantly, increasing evidence suggests that leukocyte-poor PRP formulations are biologically more favorable for ligament and intra-articular applications. Leukocyte-poor PRP has been shown to induce a lower inflammatory response, reduce the release of catabolic cytokines, and promote a more regenerative healing environment compared with leukocyte-rich PRP, which may be associated with excessive inflammation and tissue degradation [20]. These properties may be particularly relevant for intra-ligamentous ACL applications, where controlled inflammation is essential to support organized tissue regeneration [21].

In light of these findings, the present retrospective study investigates the clinical and radiological outcomes of a stem cell–based and PRP-based regenerative treatment protocol for traumatic ACL ruptures in a single-center cohort. To the best of our knowledge, this represents one of the first case series worldwide evaluating the feasibility and twelve-month outcomes of non-surgical ACL regeneration through targeted biological stimulation with autologous blood-derived stem and progenitor cell preparations.

## Materials and Methods

This study was designed as a single-center retrospective case series evaluating a biologic, non-surgical treatment protocol for traumatic anterior cruciate ligament (ACL) ruptures. During a six-month inclusion period from April to October 2024, 143 patients were screened for eligibility. Of these, 47 consecutive patients (24 males and 23 females) with an isolated traumatic anterior cruciate ligament (ACL) rupture who opted for non-operative management after comprehensive medical counseling were enrolled. Detailed screening logs were not available due to the retrospective design, which represents a limitation of the present study.

Clinical examination included standardized assessment of knee stability using the Lachman test, the anterior drawer test, and the pivot shift test. Patients with documented positive clinical findings underwent magnetic resonance imaging (MRI) to confirm the diagnosis of a complete anterior cruciate ligament rupture. In addition to confirming rupture completeness, MRI was used to assess rupture morphology, with particular focus on the integrity of the synovial sheath. Eligibility criteria included an ACL rupture confirmed by clinical examination and MRI. Patients were excluded if they presented with relevant concomitant knee pathology requiring surgical intervention, specifically including grade III meniscal tears, grade III collateral ligament injuries, or associated fractures of the distal femur or proximal tibia. The final cohort comprised 47 patients with primary (n = 44) ACL ruptures or re-ruptures of the ACL (n = 3).

All patients provided written informed consent prior to undergoing the described biologic treatment protocol.

All patients were available for clinical follow-up, and no patient was lost to follow-up during the 12-month observation period.

Pain intensity was assessed using the visual analogue scale (VAS) at 4 weeks, 3 months, 6 months, 9 months, and 12 months after treatment. Functional outcomes were evaluated using validated patient-reported outcome measures, including the International Knee Documentation Committee (IKDC) subjective knee evaluation form and the Lysholm knee score. These functional scores were recorded at 3, 6, 9, and 12 months after the intervention. In addition, time to return to sport-related activities was documented, including return to indoor cycling, running, outdoor cycling, and pivoting sports. Clearance for cycling was given on the day after the intervention. Return to subjective pre-injury activity level was recorded as a binary outcome (yes/no) at the follow-ups. All outcome measures were collected prospectively during scheduled follow-up visits.

Ligament morphology and healing progression were assessed by proton density fat-suppressed (PDFS) MRI sequences at 3 months after treatment. Synovial sheath integrity was defined as a clearly identifiable, continuous perifascicular sheath surrounding the ACL fibers on pretreatment PDFS MRI; patients with disrupted or absent sheath continuity were classified as sheath-disrupted. All MRI examinations were evaluated by a single musculoskeletal radiologist with more than 30 years of experience in knee imaging, who was blinded to clinical outcomes and treatment timeline. MRI examinations were evaluated for continuity of the ACL fibers, signal intensity, and overall ligament appearance. Due to the single-reader design without inter-rater reliability assessment, this represents a methodological limitation of the present study.

All procedures were performed in an outpatient setting. Patients were placed in a supine position. A total of 30 mL of peripheral venous whole blood was collected from the cubital vein under sterile conditions. The blood sample was processed immediately by centrifugation using a dedicated cell-processing system (Smart-M-Cell^®^, Miracell, South Korea). This process yielded 8 mL of an autologous blood-derived stem cell preparation (BSC), comprising approximately 2–3 mL of a concentrated mononuclear cell-enriched fraction suspended in 6 mL of autologous platelet-containing plasma from the same separation chamber. The remaining fractions were discarded. Based on manufacturer characterization data (GBSA Validation Study, 2021; n = 15; Smart-M-Cell^®^ System, generation M-CELL2), the BSC preparation contains a mean CD34 + hematopoietic stem cell concentration of 11.22 ± 4.7 cells/μL, a mononuclear cell proportion of 70.48 ± 10.64%, and a cell viability of 99.5 ± 0.2%. Growth factor concentrations include PDGF-AB at approximately 24,970 ± 21,404 pg/mL, VEGF at 425.51 ± 499.71 pg/mL, and EGF at 567.19 ± 665.9 pg/mL. The preparation is leukocyte-poor, with a granulocyte proportion of 29.52 ± 10.64%, substantially reduced compared to whole blood (58.40 ± 6.17%) [24].

All procedures involving the knee were performed in a strictly sterile setting. After thorough skin disinfection, the knee was sterilely draped. Local anesthesia was administered using 5 mL of 2% lidocaine. Under continuous ultrasound guidance using a handheld ultrasound scanner (Clarius™, Canada), the biologic preparations were injected directly into the torn ACL via the anterolateral parapatellar soft spot. Ultrasound guidance was used to visualize the ACL fibers, confirm needle placement, and ensure accurate delivery of the biologic agents into the injured ligament tissue.

Following the intervention, patients were allowed immediate full weight-bearing without the use of crutches or a knee brace. After the procedure, sterile adhesive dressings were applied to the injection site, and the knee was temporarily covered with a compressive bandage. Patients were instructed that the bandage could be removed on the same day once they had returned home. Routine thromboprophylaxis was not administered. Return to straight-line running was permitted from day 7 onwards, provided subjective knee stability was achieved. Patients were advised to avoid jumping and stop-and-go activities for four weeks and to refrain from pivoting sports for at least three months. All other daily activities and movements within a subjectively pain-free or low-pain range were permitted as tolerated.

All patients participated in a standardized physiotherapy program consisting of ten supervised sessions focusing on the restoration of full range of motion, neuromuscular control, lower extremity strength, and confidence in the injured knee. Physiotherapy was conducted by external physiotherapists experienced in knee rehabilitation and explicitly recommended by the treating center. Progression of rehabilitation and return to sporting activities were guided by individual clinical recovery, functional performance, and patient-reported symptoms, rather than by a fixed time-based protocol.

Statistical analysis was performed using descriptive statistics. Continuous variables are presented as mean values with corresponding 95% confidence intervals. Changes in pain and functional outcome scores over time were evaluated descriptively. No formal hypothesis testing was performed due to the uncontrolled design of the study.

## Results

A total of 47 patients (24 males, 23 females) with isolated traumatic anterior cruciate ligament ruptures were included in this retrospective case series. The mean age of the study cohort was 28.1 years (95% confidence interval [CI] 26.0–30.2). All patients completed the full 12-month follow-up period, and no patient was lost to follow-up.

Pain intensity, assessed using the visual analogue scale (VAS), showed a continuous decrease over the course of follow-up. At the 4-week follow-up, VAS scores were heterogeneous across the cohort, reflecting variable early post-treatment symptom levels. However, pain levels declined progressively at subsequent assessments. By 12 months after treatment, mean VAS scores had decreased to 0.36 (95% CI 0.19–0.53), indicating minimal residual pain in the vast majority of patients. Only one patient reported persistent symptoms and subsequently underwent secondary ACL reconstruction.

Functional knee outcomes demonstrated consistent and progressive improvement throughout the 12-month observation period. The mean International Knee Documentation Committee (IKDC) subjective score increased steadily across follow-up intervals and reached a mean value of 93.6 at 12 months (95% CI 92.4–94.8). Similarly, the Lysholm knee score showed a comparable trajectory, with a mean score of 94.0 at final follow-up (95% CI 92.7–95.3).

In a subgroup analysis based on synovial sheath integrity, patients with a preserved synovial sheath demonstrated a markedly faster return to pivoting sports compared with those without sheath preservation. In the synovial sheath–intact group (n = 15), the mean time to return to pivoting sports was 26.8 weeks (95% CI 24.9–28.7), whereas patients without an intact synovial sheath (n = 32) required a mean of 33.2 weeks (95% CI 30.9–35.5) to resume pivoting activities. In addition to the shorter recovery time, the sheath-intact group exhibited a narrower confidence interval, indicating a more consistent and predictable rehabilitation course. Detailed baseline characteristics of the two subgroups were not available due to the retrospective design, and this comparison should therefore be regarded as hypothesis-generating rather than conclusive.

These results indicate near-complete functional restoration in all but one patient, who did not respond sufficiently to the biologic treatment protocol and subsequently underwent surgical ACL reconstruction (ACLR).

Table 1 presents the complete clinical outcome data at all predefined follow-up time points.

**Table 1:** Clinical outcome measures at all follow-up time points. Values are presented as mean (95% CI)

Outcome Measure	4 Weeks	3 Months	6 Months	9 Months	12 Months
VAS (0–10)	3.36 (2.92–3.80)	2.36 (2.01–2.72)	1.85 (1.46–2.24)	1.40 (1.05–1.76)	0.36 (0.19–0.53)
IKDC (0–100)	—	79.42 (77.20–81.64)	83.38 (81.13–85.64)	89.31 (87.77–90.85)	93.61 (92.36–94.86)
Lysholm (0–100)	—	81.64 (78.95–84.33)	88.81 (86.87–90.75)	90.26 (88.23–92.28)	94.00 (92.84–95.16)

Figure 1 illustrates the distribution and progression of IKDC, Lysholm, and VAS scores of all patients (synovial sheath intact + synovial sheath not intact) over time using boxplots.

**Fig. 1:**
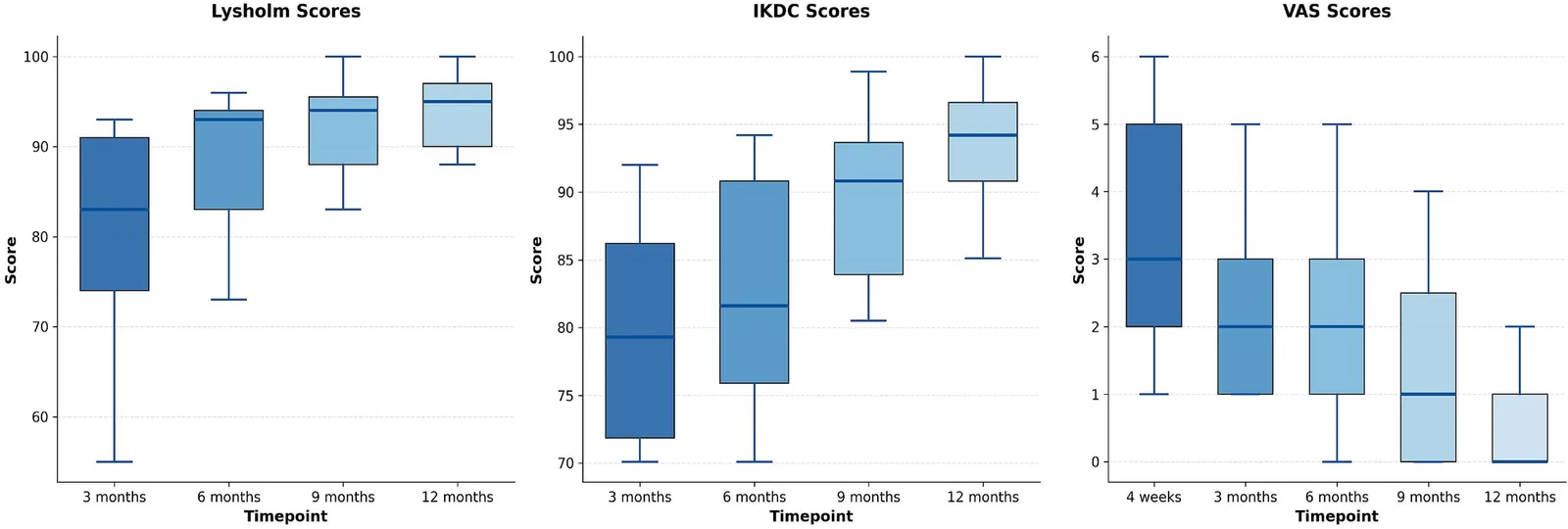
Boxplots of Lysholm, IKDC, and visual analogue scale (VAS) scores at predefined follow-up time points. Boxes represent the interquartile range with the median, and whiskers indicate minimum and maximum values

Return to sport-related activities was documented for all patients. Low-impact activities such as indoor cycling were resumed early, followed by progressive return to higher-demand activities including running and outdoor cycling.

Return to pivoting or stop-and-go sports occurred later and was guided by individual clinical recovery and functional performance. By the 12-month follow-up, all but one patient had returned to their subjective pre-injury level of sporting activity. The patient who did not demonstrate satisfactory clinical or radiological improvement subsequently underwent operative ACLR. In all but one patient, MRI findings showed evidence of improved ligament morphology, including improved fiber alignment and more homogeneous signal intensity compared with baseline imaging obtained after the index injury. It must be noted that MRI appearance alone does not confirm mechanical integrity or true histological healing.

Representative MRI images illustrating the morphological transition from a complete ACL rupture to a ligament with improved morphology on MRI are shown in Fig. 2a,b and 3a,b.

**Fig. 2:**
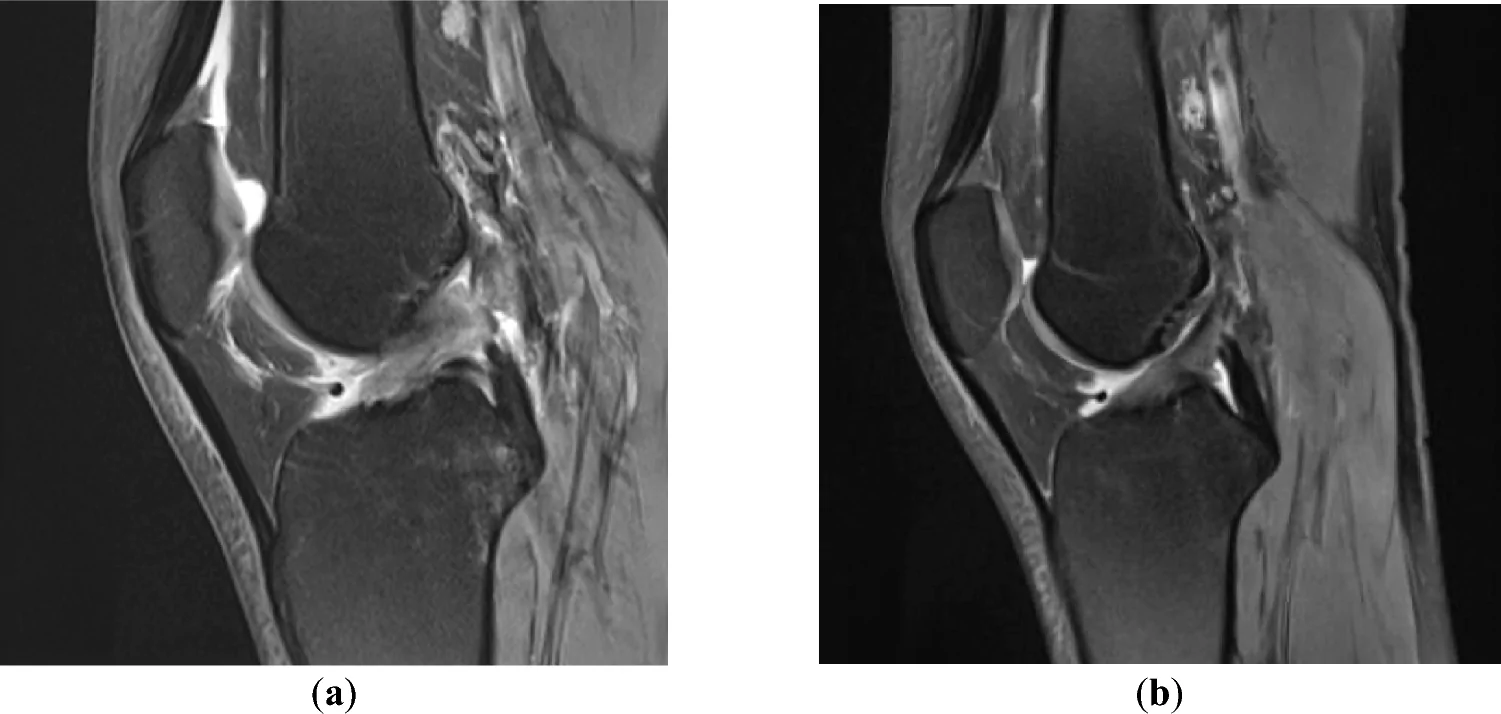
**a** Sagittal MRI of a 29-year old male patient showing a complete acute ACL rupture. **b** Sagittal MRI of the same patient at three-month post treatment demonstrating improved ACL morphology on MRI

**Fig. 3:**
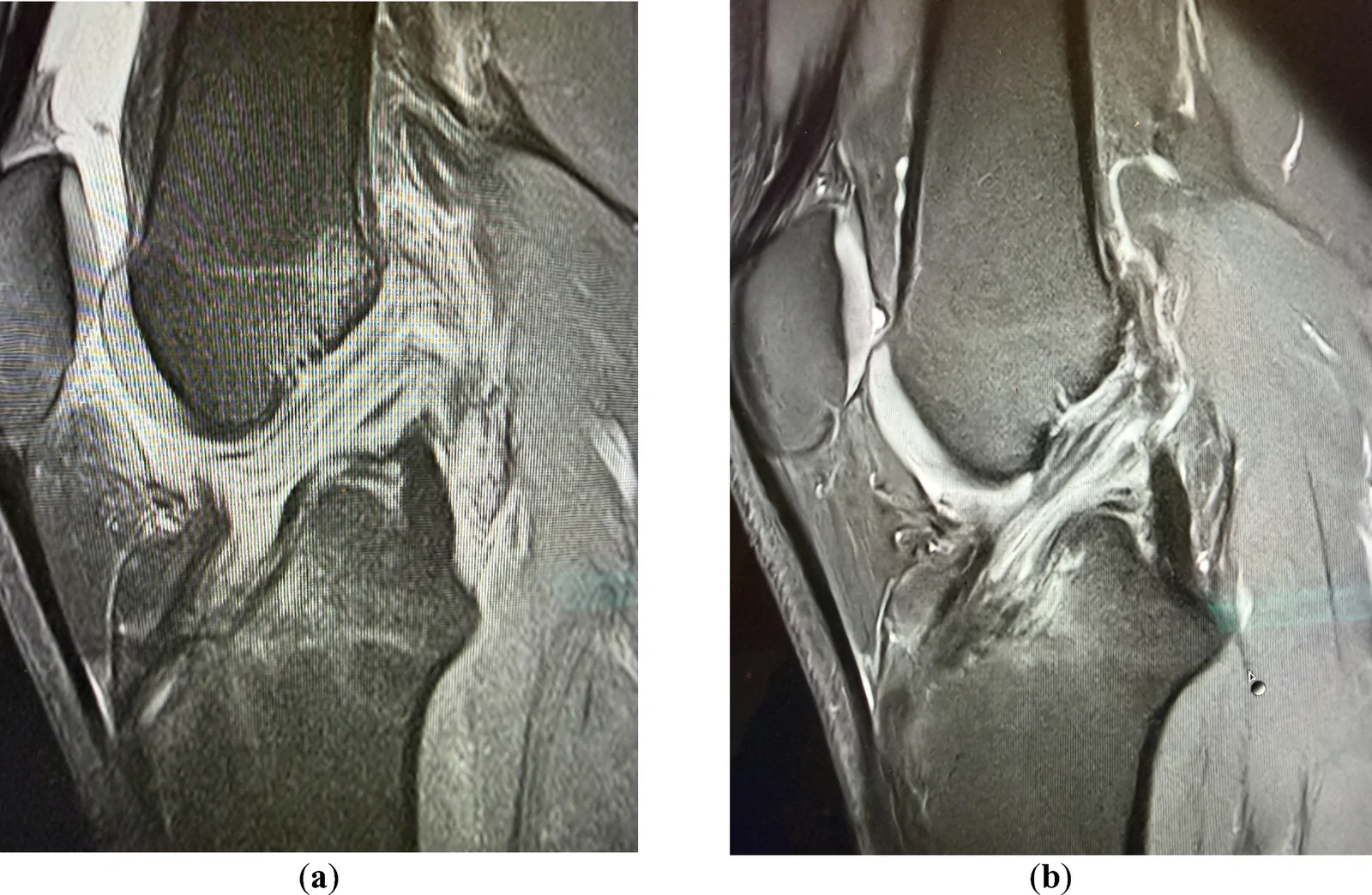
**a** Male 24-year-old professional soccer player who sustained a recurrent ACL injury. Figure shows major effusion, the ACL graft is torn, the fibers are disintegrated, the signal intensity is poor. The patient had a grade 3 anterior instability and positive pivot shift. **b** the same patient three months after treatment. The fibers of the ACL graft are well aligned and homogeneous, the signal intensity substantially improved. The patient presented with a stable knee at follow-up

No major complications or adverse events were observed during the study period. Specifically, there were no infections, thromboembolic events, neurovascular complications, or other procedure-related safety concerns. No ACL re-ruptures occurred during the 12-month follow-up period. These safety observations are limited to the 12-month follow-up period and the present uncontrolled cohort, and should not be extrapolated as evidence of broad procedural safety beyond this context. All patients completed the prescribed physiotherapy program as planned.

## Discussion

In this single-center case series, all but one of 47 patients with anterior cruciate ligament ruptures demonstrated consistent clinical improvement following a minimally invasive biologic treatment protocol combining an autologous blood-derived stem and progenitor cell preparation (BSC). Functional outcome scores (IKDC and Lysholm) improved substantially over the 12-month follow-up period, accompanied by a marked and sustained reduction in pain levels as assessed by the visual analogue scale (VAS). Importantly, the vast majority of patients returned to their pre-injury level of sporting activity, no re-ruptures were observed, and only one secondary ACLR was required during follow-up. These findings are presented as preliminary observational evidence and are not intended to demonstrate efficacy; an uncontrolled retrospective design cannot exclude natural recovery as a contributing factor.

The observed clinical and radiological improvements support the hypothesis that certain ACL ruptures retain an intrinsic biological capacity for healing when appropriate biologic stimulation is applied directly to the injured ligament. This challenges the traditional paradigm that complete ACL ruptures are incapable of healing and inevitably require surgical reconstruction. Previous MRI-based studies have demonstrated that spontaneous healing of complete anterior cruciate ligament (ACL) ruptures can occur. Under favorable biological conditions—particularly when ligament continuity and the synovial sheath are preserved—the likelihood of spontaneous healing has been reported to be as high as 30%, as described by Costa-Paz et al. [5]**.** The radiological findings in the present cohort, showing evidence of improved ligament morphology and more homogeneous fiber alignment in most patients, are in line with these observations and further support the concept that biologically mediated healing of the native ACL may be possible in selected cases [22]. From a biological perspective, the observed clinical and radiological improvements may be explained by the combined paracrine effects of the autologous BSC preparation, including its hematopoietic stem and progenitor cell fraction and platelet-derived growth factors [20, 23]. MSCs are increasingly recognized not primarily for their differentiation capacity, but for their ability to modulate inflammation, promote angiogenesis, stimulate collagen synthesis, and enhance extracellular matrix remodeling through trophic signaling mechanisms [9, 10]. Experimental studies have shown that MSCs can influence ligament healing by improving collagen organization and restoring native tissue architecture [11]. The addition of PRP may further augment this regenerative milieu by providing a concentrated source of growth factors that support cell recruitment and tissue remodeling.

Early clinical investigations of biologically augmented ACL repair strategies have reported encouraging results. MSC-based and scaffold-supported repair techniques have demonstrated functional outcomes and structural integrity comparable to surgical reconstruction in selected patient populations [13, 14]. The present study adds to this growing body of evidence by demonstrating that a purely injection-based, non-surgical approach combining BSC may achieve meaningful clinical recovery and radiological signs of ligament regeneration without graft harvesting, tunnel drilling, or hospital admission. This represents a relevant conceptual shift toward tissue-preserving, biologically oriented treatment strategies. A notable feature of the present protocol is the early timing of biologic intervention after injury and the direct intraligamentous application under ultrasound guidance. Early intervention may be critical, as the acute post-injury phase is characterized by an inflammatory and biologically active environment that may be more receptive to regenerative stimulation. Furthermore, ultrasound-guided delivery allows precise placement of biologic agents within the torn ligament, potentially enhancing local retention and efficacy compared to intra-articular injection alone.

Despite these encouraging findings, several limitations must be acknowledged. The present study is limited by its retrospective design and the absence of a control group. Because spontaneous healing of ACL ruptures has been documented [5], it cannot be excluded that natural recovery contributed to the observed improvements in some patients. The cohort was restricted to patients with isolated ACL ruptures without significant concomitant pathology, and the results may therefore not be generalizable to chronic injuries or multiligamentous knee trauma. Objective anterior laxity measurements—such as KT-1000/KT-2000 arthrometry and standardized Lachman and pivot-shift grading at follow-up—were not systematically documented, which represents a significant limitation, as the absence of objective stability data prevents definitive conclusions regarding mechanical ACL function. The standardized physiotherapy program represents a significant potential confounder, as functional recovery may be attributable in part to rehabilitation rather than the biologic intervention. In addition, although short-term functional recovery was excellent, the long-term durability of this biologically oriented, non-surgical treatment approach remains unknown.

Long-term follow-up studies after ACL injury and reconstruction have demonstrated that functional limitations and degenerative joint changes may persist or develop many years after the initial injury, regardless of treatment strategy [3]. While long-term data from the present cohort are not yet available, there are mechanistic and indirect arguments to suggest that native ACL preservation may carry advantages over surgical reconstruction with respect to osteoarthritis risk. ACL reconstruction has been consistently associated with early degenerative joint changes, persistent proprioceptive deficits, and altered joint kinematics despite restoration of mechanical stability [3, 4]. In contrast, biological healing of the native ligament—if achievable—theoretically preserves the original ligament architecture, native mechanoreceptor distribution, and physiological insertion site geometry, all of which may contribute to more favorable long-term joint homeostasis. Furthermore, avoidance of tunnel drilling, graft harvesting, and the associated inflammatory response may reduce the catabolic burden on articular cartilage. These considerations, while speculative in the absence of long-term follow-up data, provide a rational biological basis for future investigations into whether biologically augmented native ACL healing may modify the natural history of post-traumatic knee osteoarthritis.

From a clinical and health-economic perspective, the described protocol offers several potential advantages. All procedures were performed under local anesthesia, without hospital admission, and without the morbidity associated with graft harvesting or surgical reconstruction. Given the high incidence of ACL injuries and the substantial costs associated with operative treatment and rehabilitation, a minimally invasive and cost-effective biologic alternative could have relevant implications for high-volume centers, healthcare systems, and insurance providers [2].

Taken together, the present data provide preliminary support for a non-surgical, biologically oriented treatment strategy in carefully selected patients with complete ACL ruptures. While the short-term clinical and radiological outcomes are encouraging, prospective randomized controlled trials with standardized imaging protocols, clearly defined patient selection criteria, and extended follow-up are required to determine the reproducibility, long-term stability, and optimal indications for this treatment approach.

## Conclusions

In this single-center case series, patients with acute ACL ruptures showed consistent clinical and radiologic improvement following an innovative and minimally invasive biologic treatment approach based on ultrasound-guided injection of an autologous blood-derived stem and progenitor cell preparation (BSC).

These findings support the concept that the torn ACL may possess the biological capacity to regenerate. Stem-cell–based biologic stimulation may, therefore, represent a feasible non-surgical alternative for selected patients with ACL ruptures. However, given the uncontrolled study design and limited sample size, prospective randomized trials with standardized imaging protocols and long-term follow-up are required to confirm the reproducibility, indications, and durability of this treatment strategy.

## Data Availability

Data sharing is not applicable to this article as no datasets were generated or analysed that can be shared without compromising patient confi dentiality. Further information is available from the corresponding author on reasonable request.

## Declarations

### Use of Generative AI

This manuscript was prepared in part with the assistance of an artificial intelligence language model (ChatGPT, OpenAI^®^). The AI tool was used to assist with language refinement and structuring of statistical summaries only. The AI tool was not used for data analysis, interpretation of results, or scientific decision-making. All authors have critically reviewed, validated, and take full responsibility for all content of the final, submitted manuscript.
